# Protective Role of Polyphenols in Heart Failure: Molecular Targets and Cellular Mechanisms Underlying Their Therapeutic Potential

**DOI:** 10.3390/ijms22041668

**Published:** 2021-02-07

**Authors:** Rami S. Najjar, Rafaela G. Feresin

**Affiliations:** Department of Nutrition, Georgia State University, Atlanta, GA 30302, USA; rnajjar1@student.gsu.edu

**Keywords:** polyphenols, flavonoids, plant-based diets, heart failure, inflammation, oxidative stress, mitochondrial dysfunction, Ca^2+^ homeostasis, survival signaling, Sirt1

## Abstract

Heart failure (HF) is a leading cause of death in the United States, with a 5-year mortality rate of 50% despite modern pharmacological therapies. Plant-based diets are comprised of a diverse polyphenol profile, which lends to their association with reduced cardiovascular disease risk. Whether a polyphenol-rich diet can slow the progression of or reverse HF in humans is not known. To date, in vitro and in vivo studies have reported on the protective role of polyphenols in HF. In this review, we will discuss the major mechanisms by which polyphenols mitigate HF in vitro and in vivo, including (1) reduced cardiac inflammation and oxidative stress, (2) reduced mitochondrial dysfunction, (3) improved Ca^2+^ homeostasis, (4) increased survival signaling, and (5) increased sirtuin 1 activity.

## 1. Introduction

Heart failure (HF) is a major cause of death in the United States. In 2013, one in nine death certificates reported death to be due to HF [1]. HF remains a major public health concern with a 5-year survival rate of 50% even with modern medical therapies. HF is characterized by diminished ejection fraction (EF), the percent of blood pumped out of the left ventricle (LV) during each contraction, and LV hypertrophy (LVH), characterized by thickening of the LV wall [2]. HF develops due to a variety of factors, of which ischemia and hypertension are major causes. In ischemia, hypoxic conditions induce cardiomyocyte apoptosis, necrosis and promote hypertrophic signaling leading to thickening and scarring of the heart [3]. In the case of hypertension, as a maladaptive response to chronically elevated LV wall stress, hypertrophy of cardiomyocytes occurs, which also leads to apoptotic signaling and scarring of the heart [4]. To manage HF clinically, the major goals are to modify contractile signaling and improve cardiac output by reducing stress on the walls of the heart [5]. However, these therapies often neglect the underlying maladaptive cellular processes that underlie HF. Plant-based diets have been associated with reduced cardiovascular disease (CVD) risk independent of physical activity [6], inflammation [7,8], and may even be a viable adjunct treatment of HF, improving EF and decreasing LV mass [9,10,11]. Indeed, the consumption of a Mediterranean diet and the Dietary Approaches to Stop Hypertension (DASH) diet, which are characterized by increased fruit and vegetable intake, is associated with reduced incidence of HF and cardiac deaths [12]. However, no interventional studies utilizing diet have been used to clinically treat overt HF with reduced EF, with only a few case studies utilizing strict plant-based diets demonstrating reversal of diminished EF and LVH (Table 1).

Plant-based diets are typically lower in saturated fat [13], which is a variable independently associated with reduced HF mortality [14], and higher in fiber, which is associated with reduced LV mass [15] and CVD mortality [16]. Plant-based diets are associated with reduced body weight [17], hypertension [18], type II diabetes [19,20] and low-density lipoproteins [21], all of which are known risk factors in the development of HF [22,23,24,25]. However, a clinically understudied component of plant-based diets in the context of HF is the polyphenol content of plants. Since polyphenols are found nearly exclusively in plant foods, plant-based diets are inherently rich in polyphenolic compounds, which have bioactive properties that can mitigate a variety of chronic diseases at the molecular level [26]. Indeed, the intake of fruits and vegetables, which are rich in polyphenols are associated with reduced CVD risk [27]. Independently, total flavonoid and lignan consumption, rich in fruits, vegetables and whole grains, are also independently associated with reduced CVD [28]. Both the DASH diet and Mediterranean diet are rich in plants and are found to be high in polyphenols [29,30,31], which may partially explain the associated reduction in HF risk [12]. For example, in a randomized study, the consumption of an Indo-Mediterranean diet classified as rich in whole grains, fruits, vegetables and nuts was associated with a reduction in sudden cardiac death and non-fatal myocardial infarction (MI) after 2 years [32]. Further, fiber-rich diets from whole-food sources are associated with reduced CVD mortality [33]. However, since fiber intake is directly proportional to plant-food intake, and considering that a significant portion of polyphenols are bound to dietary fiber and require microbial liberation [34,35,36,37], increased fiber intake is inherently tied to increased polyphenol intake. Thus, dietary patterns which are associated with reduced CVD risk are also associated with increased plant-food intake, and correspondingly, increased polyphenol intake.

**Figure 1:**
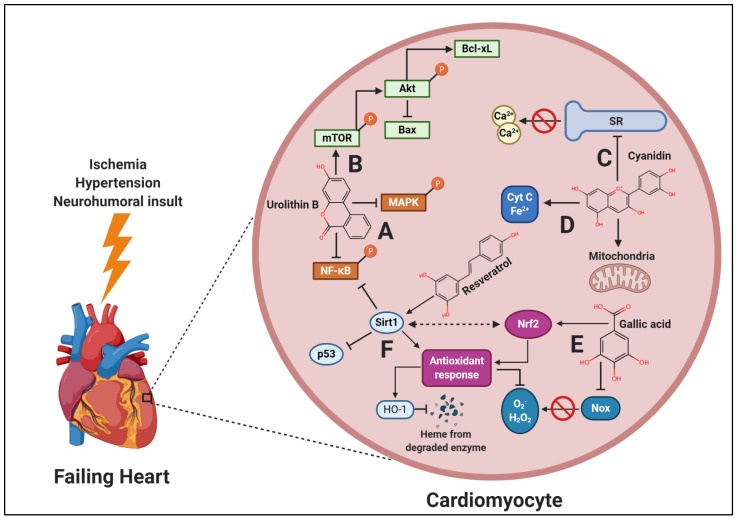
Mechanisms by which polyphenols, derived from plant-food consumption, may attenuate heart failure (HF). Under chronic, pathological conditions, HF occurs due to a variety of compensatory cellular processes. Polyphenols have many overlapping targets which can attenuate these processes in the following manner: (**A**) polyphenols, such as the ellagic acid metabolite urolithin B, reduce inflammation by decreasing nuclear factor kappa-light-chain-enhancer of activated B cells (NF-κB) and mitogen-activated protein kinase (MAPK) phosphorylation, preventing pro-apoptotic signaling and inflammatory cytokine release; (**B**) polyphenols also increase mammalian target of rapamycin (mTOR) and Akt, which reduces autophagy, apoptosis, and bcl-2-associated X protein (Bax) while increasing B-cell lymphoma extra-large protein (Bcl-xL); (**C**) Ca^2+^-dependent and calmodulin-dependent protein kinase II, calpain and calcineurin activity are attenuated by polyphenols, such as the anthocyanin cyanidin, and Ca^2+^ sarcoplasmic reticulum (SR) leak is reduced, thus normalizing cellular Ca^2+^ flux; (**D**) polyphenols act as substrates of the electron transport chain (ETC), improving ETC efficiency. They also increase the reductive state of cytochrome C (Cyt c) and prevent mitochondrial permeability transition pore opening, maintaining membrane polarization; (**E**) polyphenols, such as the phenolic acid gallic acid, reduce NADPH-oxidase (Nox) expression and increase antioxidant and detoxifying enzyme activity. This occurs due to increased nuclear factor erythroid 2-related factor 2 (Nrf2) nuclear translocation, as well as increased crosstalk between Sirt1, leading to attenuation of excessive reactive oxygen species and free heme, thus, oxidative stress and cytotoxicity is reduced. Lastly, (**F**) sirtuin 1 (Sirt1) is upregulated by polyphenols, such as the stilbene resveratrol, and also by Nrf2 activity which correspondingly increases endogenous antioxidant activity, inhibits pro-apoptotic p53, and inhibits inflammatory signaling of the NF-κB complex via deacetylation. Cumulatively, these multiple and overlapping targets of polyphenols present a potential therapeutic treatment of HF using a plant-based diet. Created with BioRender.com.

Illustrative of the efficacy of plants, investigations utilizing plant-based diets are some of the only interventional studies to clinically demonstrate regression of coronary atherosclerotic plaque [38,39]. However, investigations which aim to examine plant-based diets or even polyphenol-rich plants in the treatment of overt HF are entirely lacking, with only a limited number of case reports demonstrating clinical efficacy (Table 1). In vitro (Table S1), ex vivo and in vivo preclinical (Table S2) studies demonstrate that polyphenols derived from plants are highly efficacious in treating HF by preventing or reversing functional and morphological abnormalities of the heart by targeting underlying cellular pathways which drive the disease-process forward. Very few studies have investigated the use of plant-based dietary approaches or polyphenol-rich plants to treat HF in both preclinical models and human studies. Although polyphenols have differing structure and biological activity, they offer cardio-protective effects in the context of HF through differing mechanisms with significant overlap. These mechanisms include (1) reduction of cardiac oxidative stress and inflammatory signaling, (2) reduced mitochondrial dysfunction, (3) improved Ca^2+^ homeostasis, (4) increased survival signaling, and (5) increased sirtuin (Sirt) 1 activity (Figure 1). Thus, the aim of this review is to summarize the major cellular targets of polyphenols in the context of HF and provide evidence for future translational research utilizing plant-based dietary approaches in humans.

## 2. Polyphenols

**Figure 2:**
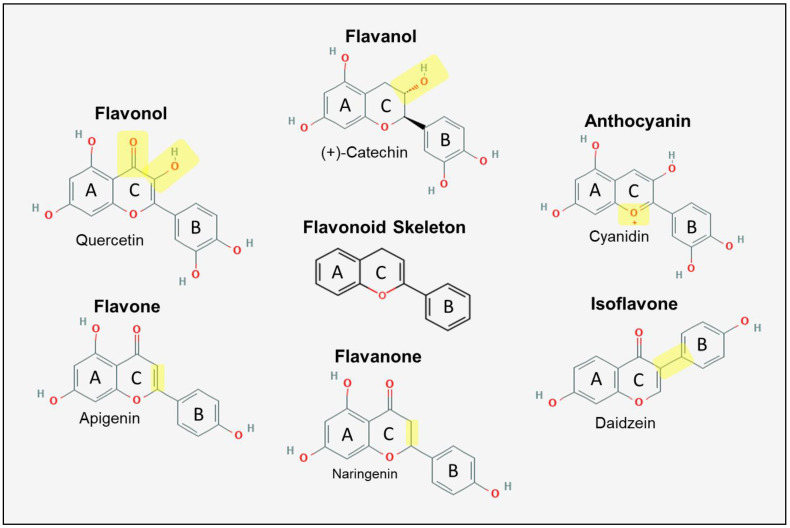
Chemical structure of common flavonoids and their classes found in edible plants. Highlighted in yellow are features which distinguish flavonoid classes from each other. Chemical structures derived from PubChem [42].

Edible plants contain polyphenols in varying concentrations (see Lorenzo et al. [40] for compiled concentrations), which can be categorized into four overarching categories: flavonoids, phenolic acids, lignans and stilbenes [41]. Flavonoids can be further subcategorized into: flavonols, flavanols, flavones, flavanones, isoflavones and anthocyanins. Flavonoids are classified based on the carbon attachment of the C ring to the B ring as well the oxidative state of the C ring as illustrated in Figure 2.

Flavonols and flavanols are the most common flavonoids found ubiquitously in most legumes, fruits and vegetables [41]. Flavonols are ketones with a hydroxyl group at position 3 of the C ring. Quercetin, kaempferol, rutin and myricetin are the most abundant flavonols. Flavanols lack the ketone of flavonols and are also called flavan-3-ols because the hydroxyl group is always at position 3 of the C ring. Flavanols exist as monomeric (catechin, epicatechin, epigallocatechin) and polymeric (proanthocyanidins, also known as condensed tannins which are simply monomeric flavanols with primarily C4→C8 bonds forms [43].

Flavones, compounds with a double bond between position 2 and 3 of the C ring and a ketone at position 4 of the C ring, are less common and typically found in peppers. Luteolin and apigenin are examples of classically studied flavones [44]. Flavanones are nearly identical in structure to flavones; however, they contain a single bond between carbons 2 and 3 of the C ring. Flavanones are typically found in citrus fruits and include hesperidin, naringenin and eriodictyol [45]. Isoflavones including daidzein and genistein are typically found in soy products and act as phytoestrogens since they have the ability to bind estrogen receptors in mammalian cells [46]. Unlike other flavonoids, the B ring of isoflavones is attached at position 3 of the C ring relative to oxygen.

Anthocyanins are derived from flavonols and have a positively charged oxygen at position 4 of C ring [47,48]. They are responsible for the blue, purple and red pigment of plant foods. Cyanidin, delphinidin, malvidin, pelargonidin, peonidin and petunidin are the most common anthocyanins found in their glycosylated form. They are referred to as anthocyanidins if they are devoid of the glycosyl moiety. Darkly pigmented plants, such as black beans [49], purple sweet potatoes [50], red cabbage [51], red lettuce [52], as well as berries [47] are known to have particularly high concentrations of anthocyanins.

**Figure 3:**
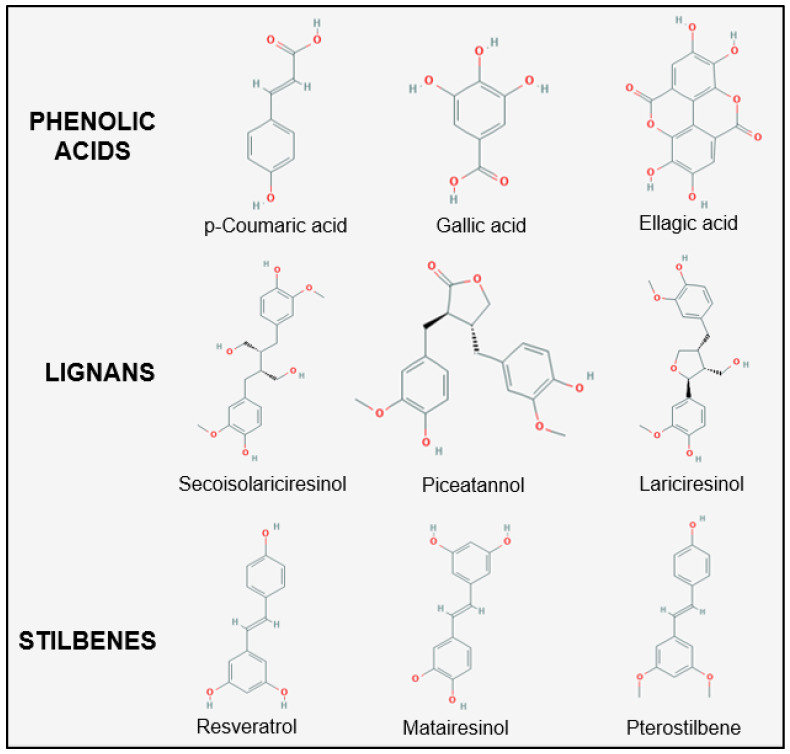
Chemical structure of common phenolic acids, lignans and stilbenes found in edible plants. Chemical structures derived from PubChem [42].

Phenolic acids contain one or more aromatic rings with a carboxylic acid and include a large number of compounds primarily classified as hydroxycinnamic acids, which have a saturated tail followed by carboxylic acid and include compounds such as caffeic, chlorogenic, ferulic and p-coumaric acid. Hydroxybenzoic acids have no tail saturation [53] and include gallic, ellagic, protocatechuic, vanillic and syringic acids (Figure 3). Phenolic acids are found nearly ubiquitously in all plant foods; however, their proportions and concentrations vary [41]. Hydrolysable tannins are high-molecular weight compounds comprised of phenolic acids, such as ellagic and gallic acid, that are released under acidic conditions in the stomach and small intestine [43]. In contrast, condensed tannins or proanthocyanidins are non-hydrolysable polymers of flavonoids including catechin and epicatechin [43].

Lignans are a separate polyphenol class and include linked diphenol compounds with two phenylpropanoids. They have differing combinations of linked lactone or carbon bonds [54] (Figure 3). Flaxseeds are one of the richest dietary sources of lignans; however, grains and nuts do contain small quantities [55]. Common lignans include secoisolariciresinol, matairesinol and lariciresinol. 

The last of the major polyphenol class includes stilbenes, the most well studied of which is resveratrol, found in high concentrations in the skin of purple grapes. However, it is also found in smaller quantities in certain blueberry varieties, strawberries, lingonberry, tomato skin and cocoa [56,57,58]. Less commonly known stilbenes contain two phenolic rings linked by an ethylene; these include piceatannol and pterostilbene (Figure 3). They are found in a variety of edible berries [57]. 

### 2.1. Polyphenol Metabolism

Parent compounds, polyphenols which have not undergone metabolic breakdown, are typically poorly absorbed in the intestinal lumen [59]. However, these parent compounds typically undergo hydrolysis, deglycosylation, dehydroxylation, and demethylation reactions to facilitate absorption, which is carried out mostly by the gut microbiota [59]. In the liver, polyphenol metabolites act as substrates for cytochrome P450, and these modified compounds are then further catalyzed by phase II enzymes and undergo glucuronidation with UDP-glucuronosyl transferases and sulfonation with sulfotransferases to increase solubility [60]. Adequate bioaccessibility of polyphenols in food facilitates availability for metabolism and absorption [40]. Mastication and changes in intestinal pH can increase bioaccessibility. Further, microbial degradation of food matrices that are indigestible to humans, namely, fiber, can liberate polyphenols for metabolism [34,35,36,37]. In fact, non-extractable polyphenols (those bound to plant matrices) comprise ~78% of the total polyphenol content of the Spanish diet [36]. 

Additionally, various cooking methods can increase or decrease bioaccessibility depending on the plant food (reviewed in detail by Lorenzo et al. [40]). For example, steaming cauliflower can increase the total polyphenol content [61], but steaming kale may decrease the total polyphenol content [62]. In a study where subjects consumed fresh tomato or tomato sauce, naringenin was not detected in the serum of subjects who consumed fresh tomatoes but appeared in those that consumed tomato sauce [63]. Naringenin glucoronide appeared in the serum of subjects that consumed tomato sauce at nine times the concentration of fresh tomatoes, while caffeic acid glucuronide appeared in serum at a greater concentration with fresh tomatoes compared to tomato sauce. In rats consuming a high-fat diet with pitaya cactus, oven cooking decreased the total phenolic content by ~37%, which was reflective in serum, as decreased antioxidant activity was observed in these animals eating oven cooked pitaya compared to fresh pitaya [64]. Whether these changes in bioaccessibility are of substantial therapeutic relevance in humans remains to be fully elucidated. 

#### 2.1.1. Flavonoid Metabolism

Non-hydrolysable tannins, such as proanthocyanidins also undergo microbial metabolism to facilitate absorption, which has been reviewed extensively by Mena et al. [65]. For example, the estimated absorption of the whole flavanol intact is low, roughly 7.5% [65]; however, following microbial metabolism, approximately 95% of flavanols may be absorbed in the form of metabolites [66]. These microbial reactions include cleavage of the inflavan bond and C-ring and a series of dihydroxylation and oxidation reactions yielding a number of metabolites, including 5-(3′,4′-dihydroxyphenyl-γ-valerolactone), 5-(3′,4′-dihydroxyphenyl) valeric acid and 2-(3′,4′-dihydroxyphenyl) acetic acid, among others [67]. Exemplifying the importance of these metabolites, the flavanol metabolite (δ-(3,4-dihydroxyphenyl)-γ-valerolactone) was found to accumulate in both human monocytes and endothelial cells and exert potent anti-inflammatory effects at low μM concentrations [68]. However, the parent compound (+)-catechin had minimal cellular uptake in vitro in EA.hy 926 cells, as demonstrated by negligible cellular antioxidant activity [69]. Interestingly, the parent compound cyanidin-3-glucoside can enter the cell by membrane transporter bilitranslocase [69], while metabolites may enter through monocarboxylate transporters [68] or even may diffuse through lipid membranes if hydroxyl groups are deficient in number [70]. In vitro in a cell-free system, the relative hydrophobicity of certain flavonoids, such as chrysin and apigenin, is higher, whereas the attachment of a glucoside typically extinguishes hydrophobic interactions [70] necessitating the use of membrane transporters or colonic metabolite generation in vivo.

#### 2.1.2. Phenolic Acid Metabolism

Phenolic acids are the most diverse polyphenols found in plant foods and are also the most common metabolite derived from the metabolism of both flavonoids and parent phenolic acids. For example, ellagitannins, polymers of ellagic acid and gallic acid, are found in high concentrations in most edible berries and nuts [71]. Following hydrolytic liberation in the stomach and small intestines, ellagic acid is metabolized by the gut microbiome to yield urolithin A, B and other urolithin derivatives, which have independent bioactive properties [72]. These urolithin metabolites can remain in serum for up to 48 h and may undergo subsequent phase II glucuronidation or sulfonation reactions even 12 h following consumption [73]. Further, phenolic acids can be derived from flavonoids; the anthocyanin cyanidin-3-glucoside is very poorly absorbed (<1% absorption) [74]. However, the phenolic acid protocatechuic acid is a metabolic byproduct of cyanidin-3-glucoside produced by bacteria in the gut, yielding a variety of other phenolic metabolites including vanillic acid, hippuric acid, caffeic acid, and ferulic acid [75]. Phenolic acids also exist unbound, some of which include chlorogenic acids, p-coumaric acids, syringic acid and gallic acid [76,77,78]. Chlorogenic acid, a hydroxycinnamic acid found in abundance in a variety of fruits, vegetables and berries [78,79,80], is hydrolyzed by gut microbes to yield caffeic acid [81]. In humans, caffeic acid is absorbed in the small intestine at three times the rate of chlorogenic acid [82].

#### 2.1.3. Lignan and Stilbene Metabolism

As with most other parent polyphenol compounds, lignans also undergo a series of dehydroxylation and demethylation reactions to yield enterodiol and enterolactone [83], of which the metabolite enterolactone is mildly associated with reduced CVD events [84]. The absorption of the common stilbene, resveratrol, is nearly zero; however, resveratrol undergoes extensive microbial metabolism, including double bond reduction, dihydroxylation, and demethylation to allow for the absorption of metabolites that are further modified in the liver [85,86]. Most resveratrol metabolites constitute resveratrol-3-*O*-glucuronide, resveratrol-4′-*O*-glucuronide, resveratrol-3-*O*-sulfate and resveratrol-4′-*O*-sulfate [87]. 

Thus, based on the metabolic fate of polyphenols, it is likely that the therapeutic efficacy of the polyphenols utilized in preclinical models is due to the molecular action of the metabolites and not necessarily the parent compounds. For example, a number of anthocyanins and flavanols with glycosides as well as ellagitannins can be found in red raspberry fruit, with polyphenol content between 71.6 and 281.0 mg per 100 g of fresh red raspberries [88]. However, following consumption of 125 g red raspberry for four weeks and 250 g red raspberry on the last and first day, pooled data indicate 62 different metabolites identified in serum, breast milk and urine, including a number of benzoic acids and urolithins derived from the metabolism of flavonoids and ellagitannins [88]. In a randomized, crossover postprandial study, 200 and 400 g red raspberry resulted in a significant improvement in endothelial function at 2 and 24 h compared to control [89]. The total concentration of polyphenols in serum at baseline was ~88 μM but increased to ~112 μM and ~119 μM at 2 and 4 h, respectively, suggesting the direct effects of polyphenol metabolites on the vasculature. In a separate investigation, the consumption of 500 mg of cyanidin-3-glucoside resulted in various peaks of hydroxybenzoic and hydroxycinnamic acids at 1, 6 and 24 h in serum with fluctuations in the nM and μM concentration range [75]. Human umbilical vein endothelial cells (HUVECs) were treated with these polyphenols at their respective serum concentrations during the differing time points in addition to 10 ng/mL of TNF-α for 24 h [90]. Even at 0.1 times the concentration of polyphenols found in serum, endothelial inflammatory protein expression of vascular cell adhesion molecule 1 and intracellular adhesion molecule 1 were significantly reduced, suggesting the potent effects of polyphenols even at sub-physiological concentrations.

## 3. Polyphenols in Heart Failure: Role of Oxidative Stress and Inflammation

Excessive cardiac oxidative stress derived primarily by overexpression of nicotinamide adenine dinucleotide phosphate (NADPH)-oxidases (Nox) and an increase in mitochondrial-derived reactive oxygen species (ROS) are major drivers of HF [91,92,93,94]. Increased ROS activates inflammatory signaling pathways including mitogen-activated protein kinases (MAPKs): p38MAPK, extracellular signal-regulated kinases (ERK)1/2 and c-Jun N-terminal kinase (JNK) [95,96,97,98], which can induce cellular apoptosis via modification functional domains of p53 [99]. Independently, ROS leads to phosphorylation and nuclear translocation of nuclear factor kappa-light-chain-enhancer of activated B cells (NF-κB) [100] in the cardiomyocyte, leading to the transcription of inflammatory cytokines including transforming growth factor (TGF)-β, interleukin (IL)-6, IL-1β and tumor necrosis factor (TNF)-α [101,102]. Inflammation facilitates macrophage recruitment into the myocardium via chemoattractants and also leads to differentiation of fibroblasts into myofibroblasts, promoting fibrosis [103]. Cumulatively, these signaling effects lead to cardiomyocyte hypertrophy, apoptosis, pro-fibrotic signaling and, at the organ level, reduced functional capacity.

To counteract the detrimental effects of oxidative stress, cellular antioxidant and detoxifying enzymes neutralize ROS and ameliorate cytotoxic conditions [104,105,106]. These enzymes include superoxide dismutase (SOD), catalase, glutathione S-transferase, glutathione peroxidase (GPx), heme oxygenase (HO)-1 and NADPH dehydrogenase quinone 1 (NQO1), which are mostly co-regulated by Sirt1 and nuclear factor erythroid 2-related factor 2 (Nrf2) [107,108,109]. In a state of exacerbated oxidative stress and inflammation as observed in HF, the detoxifying system is overwhelmed, as NF-κB overexpression can inhibit Nrf2 nuclear activity, and vice-versa [110,111]. Thus, mediating the inflammatory and antioxidant response is of major therapeutic relevance in HF.

### 3.1. Flavonoids

Of the flavonoids, flavanols, flavonols, anthocyanins and flavanones are the most commonly consumed and ubiquitously found in edible plants [112,113]; however, flavones and isoflavones are also of dietary significance and may be cardio-protective in HF. In H9c2 cardiac myoblasts treated with 5 μM of the flavanol catechin, anthocyanidins cyanidin and delphinidin, and the flavonol quercetin under hypoxic conditions to mimic ischemia, cell viability was improved compared to control cells [114]. Additionally, under conditions of oxidative stress with 400 μM tert-Butyl hydroperoxide (tBuOOH) for 24 h, 1 h pretreatment with 25 μM epigallocatechin gallate was cytoprotective, whereas 3-day pretreatment was ineffective. However, under both 1 h and 3-day pretreatment conditions with quercetin, cell survival was 95% and 66%, respectively, following tBuOOH stimulation. These data suggest the differing cytoprotective roles of flavonoids under differing treatment conditions, likely due to direct cellular antioxidant effects as well as secondary effects by increasing detoxifying enzymes to neutralize ROS. Further, H9c2 myoblasts were pretreated for 25 h with 10 ng/mL of lingonberry extract, a rich source of anthocyanins including cyanidin derivatives: cyanidin-3-galactoside, cyanidin-3-arabinoside and cyanidin-3-glucoside [115]. Following pretreatment, 600 μM of H_2_O_2_ was added to cell culture to induce apoptosis. All three anthocyanins, cyanidin-3-galactoside, cyanidin-3-arabinoside and cyanidin-3-glucoside independently prevented cell apoptosis, as did the lingonberry crude extract. 

Illustrative of these antioxidant effects, primary rat cardiomyocytes were pretreated for 45 min with 6.55 µg/mL of total flavonoids or blueberry anthocyanin extract followed by 0.25 µM norepinephrine stimulation for 24 h [116]. Norepinephrine targets the β-adrenergic receptor (β-AR), which is overstimulated during times of decreased cardiac output, a compensatory mechanism to increase cardiomyocyte excitation and contraction [5]. Overstimulation can cause cardiomyocyte toxicity. In fact, transgenic animal models in which β-AR is increased by 15-fold show a progressive decline in heart function with EF reduced to 20%, which causes overt HF [117]. In the aforementioned study [116], cardiomyocyte hypertrophy and apoptosis was increased following norepinephrine treatment; however, these changes were attenuated with flavonoid and blueberry anthocyanin extracts. Norepinephrine treatment also increased total oxidative stress and decreased SOD and catalase activity, which were reversed by blueberry crude extract (isolated fractions were not used). Additionally, in neonatal rat cardiomyocytes, pretreatment with 50 μM delphinidin for 30 min followed by 24 h treatment of 1 μM angiotensin (Ang) II, an inducer of oxidative stress via the angiotensin type-1 receptor (AT_1_R), significantly reduced both H_2_O_2_ and O_2_^•−^, which was reflective of reduced Nox activity, particularly Nox2 [118]. Interestingly, Ang II-mediated hypertrophy was attenuated by delphinidin, which was reflective of reduced activation of ERK1/2, p38MAPK and JNK. In conjunction with this in vitro model, transverse aortic constriction (TAC) in mice results in pressure overload, an acute model of hypertension-induced HF. Delphinidin, at a dose of 15 mg/kg/day delivered via intraperitoneal injection for eight weeks following TAC, preserved EF, reduced cardiac collagen accumulation, cardiac hypertrophy, O_2_^•−^ production and Nox activity. It is likely that AMP-activated protein kinase (AMPK), a cardio-protective enzyme [119], is downregulated due to TAC, which is reversed by delphinidin [118]. Upregulation of AMPK leads to inhibition of GTP-binding protein Rac1, a key regulatory subunit of both Nox1 and Nox2 [120].

While these flavonoids may be protective as they reduce oxidative stress, which thereby reduces inflammatory signaling [121,122,123], it is likely that flavonoids reduce inflammation in an ROS-independent manner. For example, the flavonol myricetin was administered to mice via gavage at a concentration of 200 mg/kg/day for six weeks following TAC, which attenuated cardiac dysfunction, hypertrophy and collagen synthesis compared to control TAC [124]. Further, Nrf2 nuclear translocation was upregulated, which corresponded with increased HO-1, decreased NF-κB nuclear translocation, and decreased MAPK signaling compared to control. Surprisingly, Nrf2 knockdown did not prevent a reduction in inflammatory signaling. In vitro, significant oxidative stress was unable to be abrogated by myricetin induced by H_2_O_2_ and Nrf2 silencing. The protective effects of myricetin are both ROS-dependent and independent, as myricetin may inhibit TAK1 by facilitating ubiquination of tumor necrosis factor receptor (TNFR)-associated factor 6 (TRAF6), just upstream of TAK1, resulting in reduced NF-κB and MAPK signaling, which are downstream of TAK1. Thus, flavonoids likely reduce oxidative stress and inflammation in a multi-targeted manner.

Flavones (luteolin and apigenin), flavanones (naringenin and hesperetin) and isoflavones (genistein and daidzein) also appear to reduce oxidative stress and inflammation in HF through similar mechanisms and pathways [125,126,127,128,129,130,131,132,133], by mediating Nrf2 and decreasing inflammatory signaling. In the case of isoflavones, it is interesting to note that this class of flavonoids is one of the only polyphenols used in clinical trials, albeit very few, and the cardioprotective effects are in moderate accordance with preclinical studies [131,132,133]. In patients with ischemic stroke, soybean isoflavone extract (55% genistein and 23% daidzein) significantly increased serum Nrf2 mRNA and SOD protein, and decreased inflammatory cytokines IL-6 and TNF-α. These changes corresponded with improved arterial function as assessed by flow-mediated dilation. Further, evidence also exists that, in subjects with metabolic syndrome, genistein improved EF compared to control subjects. These effects are particularly profound because both control and genistein treatment groups followed a Mediterranean diet and exercised as part of the intervention; however, control subjects did not improve functional parameters of the heart. Based on the presented evidence thus far in addition to numerous other in vitro and in vivo models [134,135,136,137,138,139,140,141,142,143,144,145] (Table S1), it is clear that flavonoids possess potent anti-inflammatory and antioxidant effects and attenuate HF.

### 3.2. Phenolic Acids

Similar to flavonoids, phenolic acids likely reduce ROS and inflammation in a multi-targeted manner. For example, rats receiving 10 mg/kg/day of vanillic acid or 20 mg/kg/day of losartan, an AT_1_R blocker, orally for 10 days had significantly greater expression of cardiac catalase, GPx and SOD with vanillic acid compared with losartan alone [146]. In a separate investigation, mice underwent TAC and were monitored for eight weeks, after which they received treatment with gallic acid (100 mg/kg/day), losartan (3 mg/kg/day), carvedilol (1 mg/kg/day), or furosemide (3 mg/kg/day) for two weeks [147]. These drugs (with the exception of gallic acid) are used in the clinical care to treat HF. Interestingly, gallic acid treatment was able to reverse both functional and morphological abnormalities associated with TAC, such as fractional shortening, LV end-systolic dimension (LVESD), end diastolic dimension (LVEDD), measures of LV function, as well as heart weight and perivascular fibrosis despite established HF. Neither AT_1_R inhibition (losartan), β-AR inhibition (carvedilol), nor reduced vascular pressure due to diuresis (furosemide) were able to reverse or blunt these pathological characteristics due to TAC. Further illustrated by Jin et al. [147], in primary rat cardiac fibroblasts, despite a 3 h pretreatment with TGF-β1 (5 ng/mL), an inducer of collagen synthesis, gallic acid treatment (unspecified concentration) for 9 h reversed collagen synthesis and myofibroblast differentiation. 

Other phenolic compounds also are of therapeutic relevance under a variety of cardiac stressors. For example, ellagic acid (20 µM) was able to completely blunt O_2_^•−^ production induced by hyperglycemic conditions (30 mM glucose) in isolated rat aortas. In addition, treatment with ellagic acid reduced Nox4 and ERK1/2 expression in human aortic endothelial cells under hyperglycemic conditions [148]. Gallic acid can also directly affect Nox expression in the heart, as Nox2 protein, as well as Nox1, Nox2 and Nox4 mRNA expression, were significantly reduced in spontaneously hypertensive rats due to gallic acid treatment (1% of water drinking water) for two weeks following TAC [149]. Ellagic acid (7.5 mg/kg and 15 mg/kg orally) pretreatment for 10 days followed by subcutaneous injection of 100 mg/kg isoproterenol (an agonist of β-AR) for 2 days resulted in a blunting of the arrhythmic and hypertrophic effects induced by isoproterenol [150]. In an ischemic model of HF using CAL, a derivative of p-coumaric acid (4-*O*-(2″-*O*-acetyl-6″-*O*-p-coumaroyl-β-d-glucopyranosyl)-p-coumaric acid) in a dose-dependent manner (15, 30 and 60 mg/kg/day) improved EF (61%, 69% and 71%, respectively, compared to 56% in control) in addition to decreasing cardiac TNF-α, IL-6 and IL-1β expression after eight weeks [151]. In a separate investigation, 0.7 mg/kg of urolithin B, a metabolite of ellagic acid metabolism, was injected subcutaneously in rats 24 and 48 h prior to ischemia-reperfusion (I/R) to induce HF [152]. While urolithin B did significantly reduce the infarct size, cardiac Nrf2 nuclear translocation was up-regulated, which corresponded with increased SOD activity and decreased malondialdehyde (a marker of lipid peroxidation) and O_2_^•−^ levels. This was hypothesized to be due to increased p62 expression, a scaffolding protein involved in a variety of cellular processes that can directly bind to the binding pocket of Keap1, a cytosolic Nrf2 sequestering protein, allowing for increased nuclear translocation [153].

Further illustrating the multiple targets of phenolic acids, cardiomyocytes treated with TNF-α alone (40 ng/mL) for 24 h were apoptotic compared to control. Nonetheless, these apoptotic effects were completely blunted by pretreatment with chlorogenic acid (1 µmol/L) for 12 h [154]. Further, NF-κB phosphorylation was increased due to TNF-α treatment; however, chlorogenic acid reduced NF-κB phosphorylation to control levels. Interestingly, an NF-κB inhibitor (QNZ) decreased cardiomyocyte apoptosis induced by TNF-α, though not to the extent of chlorogenic acid. It was observed that chlorogenic acid was able to reduce phosphorylation of JNK, which was primarily driving apoptosis. Chlorogenic acid also appears beneficial in attenuating hypertrophy in vivo due to MI [155] and in vitro due to β-AR agonist [156]. Thus, phenolic acids can reduce both inflammation and oxidative stress in an ROS-dependent and -independent manner.

### 3.3. Lignans

In vitro, enterolactone increases Nrf2 activity under basal conditions in a dose-dependent manner in HUVECs [157]. However, limited preclinical studies exist that assess lignans and their metabolites in traditional HF models, with a primary and secondary analysis identified utilizing I/R in vivo, one cell culture iron overload model, and another using pulmonary arterial hypertension to induce right ventricle dysfunction [158,159,160,161]. These limited investigations suggest that lignans are cardio-protective by decreasing cardiac oxidative stress and inflammation. Nonetheless, further investigations in acute HF models are needed.

### 3.4. Stilbenes

Of the stilbenes, resveratrol is the most widely studied in HF. In HUVECs, resveratrol under basal conditions significantly increases GPx1 and SOD1 mRNA and decreases Nox4 mRNA in a dose-dependent manner (1, 10, 30, 60 and 100 µM) after 24 h [162]. In primary cardiomyocytes exposed to high-glucose conditions (30 mmol/L) for 12 h, significantly increased inflammatory cytokines were detected in medium from cardiomyocytes, MAPK proteins (p38MAPK and ERK1/2) were increased, and nuclear NF-κB expression increased. However, these effects were significantly attenuated by 1-h pretreatment with 20 μmol/L of resveratrol [163]. In an isoproterenol model of HF in BALB/c mice, 100 mg/kg of resveratrol was injected subcutaneously for two weeks and concurrent isoproterenol injection occurred during the final week (50 mg/kg) [164]. Resveratrol treatment significantly reduced cardiac inflammatory cytokine protein expression, as well as mRNA of monocyte chemoattractant protein (MCP)-1. In addition, treatment with resveratrol significantly attenuated the infiltration of macrophages in the heart, a key process in the formation of collagen and scaring [165,166], which was accompanied by a significant reduction in cardiac collagen accumulation. Despite promising in vitro and in vivo findings from injected resveratrol, due to extremely low intestinal absorption of the intact stilbene [86], animal models utilizing ingested resveratrol are far more physiologically relevant due to microbial metabolism of the stilbene.

In a TAC model of HF in C57BL/6 male mice, resveratrol was provided by oral gavage (10 mg/kg) for 28 days following TAC surgery [167]. Compared to control animals which underwent TAC, resveratrol treatment significantly improved EF, reduced cardiac hypertrophy, collagen accumulation, macrophage infiltration, and cardiomyocyte apoptosis. It is interesting to note the 10-fold lower concentration of resveratrol used in this trial compared with Li et al. [164] who utilized subcutaneous injections of isoproterenol to induce HF, despite illustrating comparable cardio-protective effects. It could be argued that the mode of HF induction could account for these differences; however, Riba et al. [168] also utilized an isoproterenol model, albeit for eight weeks, with dietary resveratrol (15 mg/kg) and found significantly reduced cardiac MAPK signaling, collagen accumulation, LV mass and improved EF. Thus, resveratrol intestinal and microbial metabolites are likely as efficacious as the parent compound. Nonetheless, comparative studies are lacking.

## 4. Role of Polyphenols in Cardiac Mitochondrial Dysfunction

Mitochondria comprise 30% of total cardiomyocyte volume [169] and at the organ level, energy demands of the heart are so high that 30 kg of ATP are consumed daily by the heart in humans [170]. Mitochondrial dysfunction is a hallmark of HF, and is characterized by excessive ROS leak, which occurs in Complex I and III of the electron transport chain (ETC) leading to opening of the mitochondrial permeability transition pore (mPTP), causing membrane depolarization, inhibition of ATP synthesis, and cytochrome c (Cyt c) release from the mitochondria [171,172,173]. This activates the caspase class of proteins, triggering apoptosis. Complex IV in the ETC is a Cyt c oxidase, transferring electrons to oxygen to form water, while also priming Cyt c for reduction in Complex III [174]. Interestingly, while Cyt c cytosolic translocation can activate caspase-3 and -9 leading to apoptosis, the oxidation state of Cyt c is of relevance, as oxidized Cyt c is a much more potent agonist of caspase activation and cleavage than its reduced form [173]. *N*,*N*,*N*′,*N*′-tetramethylphenylene-1,4-diamine (TMPD), a Cyt c reductase, significantly attenuated caspase activation, which ultimately attenuated cardiomyocyte apoptosis in ischemic, isolated rat hearts [175]. 

Similar to TMPD, polyphenols themselves may act as a Cyt c reductase. For example, anthocyanins, 40 μM delphinidin 3-glucoside and cyanidin 3-glucoside significantly increased the reduction state of Cyt c in a cell-free system maximally by 78% and 50%, respectively, after 6 min. [176]. However, 6-min incubation with 40 μM pelargonidin-3-glucoside, malvinidin-3-glucoside and peonidin-3-glucoside only marginally increased the reduction state of Cyt c by 12%, 21% and 14%, respectively. In cardiomyocytes isolated from rat hearts that were perfused with or without 20 µM of cyanidin 3-glucoside and then underwent ischemia for 45 min, Cyt c translocation was significantly increased in the cytosolic fraction of both treated and untreated hearts. However, caspase activity was reduced to nearly non-ischemic levels due to cyanidin 3-glucoside eliciting Cyt c oxidative reduction. 

In a separate investigation, mitochondria isolated from rat hearts treated with delphinidin 3-glucoside and cyanidin 3-glucoside that had undergone 45 min of ischemia were able to improve state 3 respiration (high ADP and Pi) in the presence of NADH-yielding proteins: malate + pyruvate or glutamate + malate, compared to ischemic mitochondria alone [177]. Based on the respiratory control index, a function of state 3 respiration over basal respiration (lacking ADP and Pi), ETC efficiency was reduced by 60% under ischemic conditions with pyruvate + malate. However, 20 µM of delphinidin 3-glucoside or cyanidin 3-glucoside treatment ameliorated this effect, improving ETC efficiency by ~55% compared to ischemic mitochondria. This corresponded to overall increased ATP production with delphinidin 3-glucoside and cyanidin 3-glucoside compared to ischemic mitochondria alone. These protective effects are likely attributable to improved complex I activity. Under ischemic conditions, Complex I activity was reduced by 59%; however, delphinidin 3-glucoside and cyanidin 3-glucoside significantly increased NADH oxidation, which approached levels of that of non-ischemic control mitochondria. Interestingly, depriving coenzyme Q_1_ resulted in negligible NADH oxidation in Complex 1 of the ETC in both non-ischemic and ischemic mitochondria. Nonetheless, delphinidin 3-glucoside and cyanidin 3-glucoside significantly increased Complex 1 activity multifold despite a lack of coenzyme Q_1_. These data suggest that delphinidin 3-glucoside and cyanidin 3-glucoside can substitute coenzyme Q_1_ as electrophiles, which improves Complex I activity, thus improving overall ETC efficiency under ischemic conditions. 

Similar protective effects were observed in cardiomyocytes pretreated with chlorogenic acid (1 µmol/L for 12 h) followed by TNF-α treatment (40 ng/mL for 24 h) [154]. While TNF-α alone increased mPTP and protein expression of cleaved caspase-3 (activated), chlorogenic acid pretreatment completely blunted these effects, which corresponded to a significant attenuation in TNF-α-induced apoptosis. In vivo, senescence-accelerated prone 8 (SAMP8) mice (mice with accelerated aging) at 10 months of age received chlorogenic acid bound to a phospholipid complex (to increase absorption) at 10 or 20 mg/kg/day for two weeks [178]. Following treatment, I/R injury was induced, after which animals were immediately sacrificed. Chlorogenic acid significantly decreased cardiac mitochondrial ROS, which corresponded to increased total SOD and GPx activity, compared to I/R alone in a dose-dependent manner. Further, isolated cardiac mitochondria from I/R mice had decreased oxygen consumption compared to control, which was ameliorated by chlorogenic acid. Additionally, cytosolic Cyt c was reduced due to chlorogenic acid not only compared to I/R control mice, but sham-operated mice as well, demonstrating cytoprotective effects even in a non-pathological state. 

Mitochondria undergo regular fission and fusion, and, in healthy cardiomyocytes, this dynamic is at equilibrium [179]. In HF, this equilibrium is disrupted, and mitochondria undergo excessive fission, leading to fragmented, dysfunctional mitochondria. Fragmented mitochondria due to excessive fission produce excessive ROS [179]. Further, Bcl-2 19-kD interacting protein 3 (Bnip3) is a pro-apoptotic protein, which can associate with mitochondria following hypoxia (ischemia) or other forms of cytotoxicity (doxorubicin) facilitating mitochondrial ROS and mPTP opening [180,181]. Ellagic acid (50 and 100 µM for 1 h) has been previously shown to prevent the inhibitory effects of bevacizumab (50 µg/mL; anti-cancer drug) on succinate dehydrogenase in rat heart mitochondria as well as preserve mitochondrial membrane potential [182]. In both hypoxic and doxorubicin (10 μM)-induced rat cardiomyocyte toxicity, 10 μM ellagic acid co-treatment for 18 h reduced mitochondrial fragmentation and Bnip3 protein expression, which corresponded with reduced mPTP, ROS and an attenuation of apoptosis [183]. Thus, polyphenols likely target mitochondria in a cytoprotective manner in a multitude of ways, including increased ETC efficiency, oxidative reduction of Cyt c, and prevention of mPTP opening.

## 5. Polyphenols in Ca^2+^ Homeostasis

Ca^2+^ in the cardiomyocyte is primarily stored in the sarcoplasmic reticulum (SR). Transient flux of Ca^2+^ from the SR to the cytosol enables excitation–contraction coupling, and contraction occurs via cross-bridge formation between myofilaments in the cell [184]. Ca^2+^-dependent and calmodulin-dependent protein kinase II (CaMKII) responds to ROS (at physiological concentrations), as well as β-AR activation [185,186]. CaMKII activation leads to phosphorylation of ryanodine receptor 2 (RYR2) on the SR, allowing calcium to be released [184]. Immediately following RYR2-mediated Ca^2+^ release, sarcoplasmic-endoplasmic reticulum Ca^2+^ ATPase (SERCA2a) sequesters Ca^2+^ back in the SR. In HF, Ca^2+^ accumulates in the cytosol due to CaMKII overexpression, as well as decreased SERCA2a activity facilitating SR leak, preventing repolarization for contraction [184]. This is further exacerbated by calpains, Ca^2+^ sensitive proteases, which degrades the SR due to excessive Ca^2+^ accumulation [187]. Calpains are also activated independent of Ca^2+^ by NF-κB activity (IκBα of the NF-κB complex binds directly to calpains), as well as direct phosphorylation of calpains by ERK [188]. Elevated Ca^2+^ leads to the activation of calcineurin, a phosphatase, which dephosphorylates calcineurin–nuclear factor of activated T cells (NFAT), which dimerizes with NF-κB and translocates to the nucleus initiating hypertrophic transcription [189,190]. Polyphenols may play a role in maintaining Ca^2+^ homeostasis via a variety of pathways.

In primary rat cardiomyocytes pretreated with 6.55 µg/mL of polyphenols extracted from blueberries for 45 min followed by 0.25 µM norepinephrine stimulation for 24 h, calpain activity was found to be significantly increased due to norepinephrine but was significantly attenuated with pretreatment with blueberry polyphenols [116]. Further, dibucaine alone, a calpain inducer, significantly induced cardiomyocyte apoptosis [116]. Interestingly, blueberry polyphenols were able to completely blunt dibucaine-induced cell death and dibucaine-induced ROS. However, the Nox inhibitor VAS-2870 did not reduce norepinephrine-induced apoptosis, suggesting that these polyphenols were acting on a calpain-specific mechanism [116]. Further, norepinephrine significantly impaired cardiomyocyte contractility as evidenced by decreased peak shortening and velocity of shortening, which was also attenuated by blueberry polyphenol treatment. In a separate investigation, H9c2 cardiomyocytes and primary rat ventricular neonatal cardiomyocytes were treated with metabolites (12 μM catechol-*O*-sulphate, 6 μM pyrogallol-*O*-sulphate and 3 μM 1-methylpyrogallol-*O*-sulphate) found in human serum following the consumption of blueberry, blackberry, raspberry and strawberry [191]. Cardiomyocytes were treated for 2 h with these polyphenols, washed, and then treated with 200 μM isoproterenol for 24 h. Interestingly, polyphenols without isoproterenol increased CaMKII phosphorylation compared to control; however, isoproterenol alone increased CaMKII phosphorylation to a significantly greater extent. Treatment with both polyphenols and isoproterenol attenuated CaMKII phosphorylation compared with isoproterenol alone. These results demonstrate attenuation in aberrant and excessive Ca^2+^ flux and asynchronous beating induced by isoproterenol, particularly with catechol-*O*-sulphate and 1-methylpyrogallol-*O*-sulphate.

Both flavonoids and phenolic acids appear to have this Ca^2+^ clearing effect. For example, serum was collected from rats with cardiac physically induced trauma (resulting in significantly increased serum TNF-α) and without trauma. Serum was added to H9c2 cardiomyocytes, of which, serum containing significant TNF-α-induced significant Ca^2+^ flux which corresponded with increased apoptosis and ROS. However, quercetin (10 μM) pretreatment for 24 h following 3 h treatment with serum containing TNF-α completely abrogated these detrimental effects [192]. In a separate investigation, cardiomyocytes were isolated from streptozotocin-induced diabetic rats treated with urolithin A or B (2.5 mg/kg/day) delivered by intraperitoneal injection for three weeks [193]. Improved Ca^2+^ clearance was observed due to urolithin treatment compared with streptozotocin alone, as was contractile function which reflected improved hemodynamic data in vivo. Additionally, direct effects on the sarcoplasmic reticulum were observed, as SERCA2a protein expression was significantly increased with urolithin treatment compared to streptozotocin alone. Further, spontaneously hypertensive rats treated with gallic acid (1% of tap water) for three months had reduced cardiac CaMKII at the proteome and transcriptional level [194]. Lastly, in neonatal rat cardiomyocytes treated with gallic acid (10 μM) after 24 h treatment with Ang II (100 nM), both calcineurin and NFAT protein expression were significantly reduced compared to Ang II alone [195]. Based on these data, polyphenols likely have direct effects on maintaining calcium homeostasis in HF, including regulation of CaMKII, calpains and calcineurin as well as maintenance of SR.

## 6. Polyphenols in the Regulation of Survival Signaling

Mammalian target of rapamycin (mTOR) is part of protein complexes mTOR complex 1 (mTORC1) and mTOR complex 2 (mTORC2). Survival signaling is mediated by mTOR [196], and genetic knockout of mTOR results in dysfunctional mitochondria, cardiomyocyte apoptosis and increased autophagy [197]. For example, mTORC1 phosphorylates unc-51-like kinase (ULK) 1, preventing the formation of the autophagosome complex, inhibiting autophagy [198]. Inhibition of chronically elevated autophagy has beneficial effects in improving cardiomyocyte survival and reducing apoptosis [199,200]. For example, TAC in mice resulted in an increase in cardiomyocyte autophagy, while inhibition of autophagosome formation attenuated pathological remodeling of the heart [201]. Further, phosphoinositide 3-kinase (PI3K) phosphorylation due to growth hormones, including insulin-like growth factor-1 (IGF-1) [202], can phosphorylate mTORC2, which in turn phosphorylates Akt [196]. Akt phosphorylation prevents apoptosis via the downstream inhibition of Bnip3 [203,204] and pro-apoptotic Bcl-2-associated X-protein (Bax), both of which translocate from the cytosol to mitochondria, disrupting mitochondrial membrane integrity, causing Cyt c release resulting in cellular apoptosis [205]. B-cell lymphoma extra-large (Bcl-xL) is also increased by Akt [206]. Bcl-xL is anti-apoptotic by disrupting the caspase-3 cascade preventing apoptosis [207]. Akt inactivation is apparent in mice that undergo TAC and it is accompanied by activation of fetal genes leading to pathological hypertrophy [208].

Interestingly, mTORC1 activation appears to act in both a beneficial and detrimental manner, and its partial inhibition may preserve its physiological functions (increased mitochondrial biogenesis and oxidative capacity of nutrients) but blunt its maladaptive response (cardiac hypertrophy). mTORC1 does indeed inhibit autophagy [198] and lysosomal activity [209] and while its genetic deletion results in a rapid progression towards HF [197], partial mTORC1 inhibition in mice that underwent TAC and CAL resulted in significantly improved cardiac function and reduced cardiomyocyte apoptosis. Yet, Akt phosphorylation was preserved compared with control mice [210]. Further, mTORC1 overexpression in a high-fat diet mouse-model of MI increases infarct size and cardiomyocyte apoptosis; decreased autophagy was implicated in these detrimental effects [211]. It is interesting to note that autophagy was protective in this model which is in direct contrast with other investigations [199,200]. However, total mTOR, which upregulates both mTORC1 and mTORC2, does not appear to induce these detrimental effects [212]. In transgenic mice which underwent TAC with overexpressed mTOR, cardiac function was preserved, and fibrosis was significantly reduced compared to wild-type mice. Additionally, transfected cardiomyocytes with upregulated mTOR in vitro challenged with lipopolysaccharide, a traditional inducer of inflammation and apoptosis, resulted in decreased NF-κB signaling and decreased inflammatory cytokine expression compared with non-transfected cardiomyocytes. Unlike mTORC1, inhibition of mTORC2 in HF does not appear protective [213], as Akt phosphorylation appears highly dependent upon mTORC2 activation [214]. Despite these complexities, which are likely attributable to the mode of HF induction [196], it can be tentatively surmised that total mTOR activation appears to be of importance in attenuating HF. 

Polyphenols likely play a direct role in the regulation of total mTOR, thus modulating its downstream effectors. For example, in a streptozotocin-induced diabetes model of HF, extracted black rice anthocyanins (72% cyanidin 3-glucoside) were provided to rats at 250 mg/kg/day for four weeks following induction of type 1 diabetes [215]. Interestingly, anthocyanins increased both cardiac IGF-1 and IGF-1 receptor, which corresponded with increased Akt phosphorylation compared with untreated diabetic rats. Concurrently, reduced Bax, cytosolic Cyt c, cleaved caspase-3 and apoptosis were observed, while, functionally, EF was improved. In an in vitro I/R model, neonatal rat cardiomyocytes were pretreated with urolithin A (10 μM) at 24 and 1 h followed by I/R (3 h hypoxia then 3 h reperfusion) [216]. There was an increase in the phosphorylation of Akt compared to I/R alone, which paralleled reduction of Bax, increased Bcl-2 and a reduction of cleaved caspase-3. The protective effects of urolithin A were ameliorated with an inhibitor of PI3K, LY294002. In a separate investigation, urolithin B were injected into mice (0.7 mg/kg) at 48 and 24 h prior to induction of I/R [152]. Phosphorylation of mTOR in cardiac tissue was reduced due to I/R but restored with urolithin B, which also paralleled ULK1 phosphorylation. Further, cleaved caspase-3 and cardiomyocyte apoptosis were reduced due to urolithin B treatment. In H9c2 cells pretreated with 20 μM urolithin B for 12 h followed by hypoxia for 3 h and 3 h of reperfusion, mTOR phosphorylation was increased, as was Akt phosphorylation and ULK1, all of which reflected reduced apoptosis. While phosphorylation of mTOR, Akt and ULK1 were ameliorated by LY294002, cleaved caspase-3 and apoptosis were not significantly increased. These effects highlight differences in polyphenol activity. For example, in vitro, urolithin A seemed to primarily mediate survival signaling in I/R [216], while urolithin B operated in a more Nrf2-dependant manner in I/R [152]. 

## 7. Polyphenols in the Regulation of Sirtuin 1

Sirtuins are a class of NAD^+^-dependent deacetylases which are localized in mitochondria, the nucleus and cytoplasm. There are seven classes of sirtuins; however, Sirt1 may be of particular relevance in HF due to modifications of various cellular targets, including p53 inhibition [217], increased AMPK [218], SOD2, catalase and GPx [219,220,221], and an attenuation of NF-κB [222]. In a cross-sectional analysis of human subjects, reduced circulating Sirt1 mRNA in leukocytes was associated with HF compared with healthy controls and was also tied to reduced serum antioxidant status and increased oxidative stress [223]. Reduced Sirt1, SOD2 and Bcl-xL as well as increased p53 and Bax protein expression were also evident in atrial myocytes extracted from HF patients compared with healthy controls [224]. 

Polyphenols likely act to increase Sirt1 in a multifaceted manner. For example, in HUVECs, quercetin increased Sirt1 mRNA under basal conditions in a dose-dependent manner (2.5, 5 and 10 µM) in 24 h suggesting transcriptional regulation of Sirt1 without stress [225]. However, polyphenols not only regulate Sirt1 expression, but also enhance protein–protein interactions between Sirt1 and its substrates [226]. For example, in both C2C12 myoblasts and neonatal rat ventricular myocytes, Ang II treatment (100 μmol/L) for 8 h significantly increased ROS and apoptosis which was ameliorated with 4 h of pretreatment with 40 and 100 µmol/L resveratrol [221]. Sirt1-siRNA completely abolished the protective effects of resveratrol. Nonetheless, resveratrol did not increase Sirt1 protein expression in the absence of Sirt1-siRNA suggesting that resveratrol was exerting its effects by improving protein–protein binding affinity between Sirt1 and substrate. In human coronary arterial endothelial cells, both 48 h pretreatment of 10 μmol/L resveratrol and transfection with Sirt1 overexpression attenuated basal mitochondrial ROS [227]. Under high glucose conditions (30 mM), resveratrol decreased mitochondrial ROS in a dose-dependent manner. This effect was abolished by Sirt1-siRNA. Similar protective effects of resveratrol have been observed in Sirt1-dependent mechanisms in vitro by inhibiting hypoxia-induced apoptosis in endothelial cells [228], as well as H_2_O_2_-induced apoptosis [229] and norepinephrine-induced hypertrophy [230] in cardiomyocytes.

Resveratrol is not exclusive in its Sirt1-mediated effects. In isolated primary rat cardiomyocytes which underwent 3 h of anoxia (95% N_2_ and 5% CO_2_) and 2 h of reoxygenation (95% O_2_ and 5% CO_2_), pretreatment with kaempferol (20 μM) for 24 h improved cell viability to such a great extent that it was near that of control cells that did not undergo anoxia [231]. However, cells which underwent anoxia without kaempferol had an ~80% reduction in viability. Co-incubation of kaempferol with 60 μM of sirtinol, a Sirt1 inhibitor, ameliorated the protective effects of kaempferol. Interestingly, protein expression of Sirt1 was significantly increased by anoxia + kaempferol treatment compared with anoxia alone which again was abrogated by sirtinol. These findings ran in exact parallel with improved mitochondrial potential, reduced mPTP, ROS, apoptosis and reduced protein expression of Cyt C, cleaved caspase-3, and increased Bcl-xL. These effects were mediated by kaempferol and Sirt1. Further, mice received 100 mg/kg/day of curcumin, via oral gavage, for one week, then underwent coronary artery ligation (CAL) and were sacrificed four weeks later [232]. Mice that received curcumin had significantly greater cardiac Sirt1 protein expression compared to animals that did not receive curcumin. Additionally, curcumin supplementation significantly reduced fibrosis accumulation in the myocardium and reduced infarct size. Similar results were observed in high-cholesterol-diet-fed rats supplemented with 100 mg/kg epigallocatechin-3-gallate; Sirt1 protein expression in the myocardium was increased, as was catalase, SOD and GPx, which corresponded with reduced neutrophil infiltration of cardiac tissue and improved muscle fiber architecture [233].

Despite the protective role of Sirt1 described thus far, extreme overexpression of Sirt1 likely has pathological effects. In six-month-old transgenic mice, a 12.5-fold increase in the overexpression of Sirt1 paradoxically resulted in mitochondrial dysfunction and detrimental effects in heart function and morphology, namely, reduced EF and LVH, characteristics of HF, compared with a 2.5–7.5-fold increase in the overexpression of Sirt1 [219]. Because Nrf2 nuclear acetylation is a necessary modification in facilitating the transcription of endogenous antioxidants, deacetylation of Nrf2 facilitated by Sirt1 prevents Nrf2 transcriptional activity and shunts nuclear Nrf2 back into the cytosol in transfected K562 cells [234]. In contrast, glomerular mesangial cells that overexpressed Sirt1, increased Nrf2 nuclear accumulation and transcriptional activity. Thus, the co-regulatory role of Nrf2 and Sirt1 may be tissue- or cell-specific. For example, in a cerebral artery occlusion animal model, Sirt1 inhibition inhibited Nrf2 and vice-versa [235]. These findings may be attributed to excessive oxidative modifications of Sirt1 due to a compromised Nrf2-mediated endogenous antioxidant response including ROS-mediated carbonylation, S-glutathionylation and S-nitrosylation of cysteine residues, inhibiting Sirt1 activity [236]. Thus, modest upregulation of Sirt1 activity mediated by polyphenols as observed thus far likely acts in a protective and not a pathological manner, maintaining Nrf2 transcriptional activity as observed in prior investigations.

## 8. Conclusions

In this review, we have highlighted multiple targets and pathways that polyphenols modulate in HF, namely, a reduction of cardiac oxidative stress and inflammation, improved mitochondrial function and integrity, preserved sarcoplasmic reticulum dynamics, and increased mTOR, Akt and Sirt1 expression. Plant-based foods contain a wide variety of polyphenols; thus, consumption of whole plant-based foods may be more efficacious than treatments utilizing single polyphenols (parent compound or metabolites). This is because the multiple polyphenols found in plant-based foods can work in a synergistic or additive manner, targeting several pathways underlying HF. To date, there is a paucity of human studies reporting on the role of plant-based foods or diets in the treatment of HF. The compelling preclinical findings summarized in this review demonstrate a need for well-controlled clinical trials utilizing polyphenol-rich plant-based foods and diets to treat HF. This would ultimately lead to the development and recommendation of complementary or alternative nutritional strategies to prevent and/or manage HF. This would be of major significance to HF patients who take an average of 6.8 prescription drugs daily [237], which put them at increased risk for drug interaction. In addition, these prescription drugs are costly and not free of side effects. Therefore, the search for natural therapeutic strategies to manage HF is warranted.

## Figures and Tables

**Table 1:** Case studies utilizing plant-based diets in the clinical treatment of heart failure accompanied by reduced ejection fraction.

Subject Characteristics	Intervention	Duration	Findings	Author
1 overweight male, 79 years of age	Plant-based diet comprised of fruits, vegetables, legumes, nuts and whole grains	2 months	↑ EF ↓ angina	Choi et al. 2017 [9]
1 obese female, 54 years of age	Plant-based diet comprised of fruits, vegetables, legumes, nuts and whole grains	5 ½ months	↑ EF	Alllen et al. 2019 [10]
1 obese female (46 years), 2 obese males (58 and 70 years of age),	Plant-based diet comprised of primarily raw fruits, vegetables and seeds with some whole grains	~79 days	↑ EF, stroke volume, cardiac output ↓ LV mass, angina	Najjjar and Montgomery, 2019 [11]

↑ denotes increase, and ↓ denotes decrease. Abbreviations: EF: ejection fraction; LV: left ventricle.

## Supplementary Materials

The following are available online at https://www.mdpi.com/1422-0067/22/4/1668/s1: Table S1: Effects of Polyphenols in in vitro models of HF, Table S2: Effects of Polyphenols in ex vivo and in vivo preclinical models of HF.
