# Supplementary material for: Protective Role of Polyphenols in Heart Failure: Molecular Targets and Cellular Mechanisms Underlying Their Therapeutic Potential
Source: Int J Mol Sci. 2021 Feb 7;22(4):1668. doi: 10.3390/ijms22041668 (PMC7914665; doi:10.3390/ijms22041668)
Supplement: Supplementary file 1 [file ijms-22-01668-s001.pdf]

Table S1. Effects of Polyphenols in *in vitro* models of HF.

Polyphenol(s)	Cells	Dosage	Duration	Insult	Findings	Author
Luteolin	Rat cardiomyocytes	16 μmol/L	18 h	Isolated from rats with established HF	↑ SERCA2a, Akt improved contractility and Ca^{2+} handling.	Hu et al. [89]
Naringenin	H9c2 cardiomyocytes	80 μmol/L	6 h pretreatment	Ischemia-reperfusion	↓ apoptosis, cleaved caspase-3, O_2^-, lipid peroxidation, Nox subunit, ↑ catalase, SOD2	Yu et al. [78]
Luteolin	Rat cardiomyocytes	2, 4, 8 or 16 μmol/L	12 h pretreatment	Ischemia-reperfusion	↓ Bax, apoptosis ↑ Bcl-2, Akt, SERCA2a improved contractility	Fang et al. [88]
Lingonberry polyphenol fractions containing: cyanidin-3-galactoside, cyanidin-3-glucoside and cyanidin-3-arabinoside	H9c2 cardiomyocytes	1:100, 1:500, 1:1000, and 1:2000 dilutions	24 h pretreatment	Ischemia-reperfusion	↓ cleaved caspase-3, apoptosis, JNK	Isaak et al. [93]
Urolithin B	H9c2 cardiomyocytes	20 μM	12 h pretreatment	Ischemia-reperfusion	↓ cleaved caspase-3, apoptosis ↑ Akt, mTOR, ULK1, Nrf2, HO-1, NQO1, GST1	Zheng et al. [106]
Urolithin A	Neonatal rat cardiac myocytes	10 μM	24 h and 1h pretreatment	Ischemia-reperfusion	↓ apoptosis, O_2^-, lipid peroxidation, Bax, cleaved caspase-3 ↑ SOD, PI3K, Akt, Bcl-2	Tang et al. [172]
Kaempferol	Rat cardiomyocytes	10, 20 or 40 μM	24 h pre-treatment	Ischemia-reperfusion	↓ ROS, Cyt C, cleaved caspase-3, apoptosis ↑ cell viability, Sirt1, mitochondrial membrane potential, mPTP, Bcl-2	Guo et al. [187]
Catechin, cyanidin, delphinidin, quercetin	H9c2 cardiomyocytes	5-50 μM	3-day pretreatment, 5 h ischemia, 18 h reperfusion	Ischemia-reperfusion and Tert-butyl hydroperoxide	↑ cell viability	Akhlaghi & Bandy [64]
Myricetin	Neonatal rat cardiomyocytes	20 μM	12 h co-treatment	Phenylephrine or H_2O_2	↓ hypertrophy. MAPK, TAK1, lipid peroxidation ↑SOD, Catalase, Nrf2, HO-1	Liao et al. [74]
Cyanidin-3-galactoside, cyandin-3-glucoside, and cyanidin-3-arabinoside	H9c2 cardiomyocytes	10 ng/mL	24 h pretreatment followed by 2 h H_2O_2	H_2O_2	↓apoptosis	Isaak et al. [65]

Hesperetin	H9c2 cardiomyocytes	10 µM	12 h pretreatment, followed by 24 h isoproterenol	Isoproterenol	↓ O_2^-<br>↑ Nrf2	Velusamy et al. [79]
Chlorogenic acid	Neonatal rat cardiomyocytes	10, 50 or 100 µM	2 h pretreatment then 48 h isoproterenol	Isoproterenol	↓ hypertrophy, NF-κB activity, ROS	Li et al. [110]
Catechol-O-sulphate, pyrogallol-O-sulphate, and 1-methylpyrogallol-O-sulphate	H9c2 cardiomyocytes and rat cardiomyocytes	12 µM catechol-O-sulphate; 6 µM pyrogallol-O-sulphate; and 3 µM 1-methylpyrogallol-O-sulphate:	2 h pretreatment followed by 24 and 48 h isoproterenol	Isoproterenol	↓ ROS, calmodulin-dependent protein kinase II<br>↑ cell viability improved beating patterns and Ca^{2+} flux	Dias-Pedroso et al. [147]
Chlorogenic acid	Cardiomyocytes from urine human induced pluripotent stem cells	0.1 µmol/L	12 h pretreatment followed by 24 h TNF-α	TNF-α	↓ apoptosis, cleaved caspase-3, Bax, NF-κB, JNK<br>↑ mitochondrial membrane potential, Bcl-2, ERK1/2	Tian et al. [108]
Genistein	Cardiac fibroblasts	20, 50 and 100 µM	24 h co-treatment	TGF-β1	↓ collagen α-smooth muscle actin, TAK1, JNK	Qin et al. [81]
Gallic acid	Neonatal cardiac fibroblast	100 µM	TGF-β1 for 3 h, gallic acid for 9 h	TGF-β1	↓ collagen synthesis	Jin et al. [101]
Blueberry flavonoids and phenolic acids	Rat cardiomyocytes	0.65-6.55 µg/mL (unspecified treatment concentrations)	45 min pretreatment, 24 h norepinephrine	Norepinephrine	↓ lipid peroxidation, apoptosis, SOD, catalase, calpain Improved contractility	Louis et al. [66]
Resveratrol	Rat cardiomyocytes	15, 30 and 60 µmol	30-min pre-incubation with resveratrol then 24 h norepinephrine	Norepinephrine	↓ hypertrophy	Thandapilly et al. [186]
Gallic acid	H9c2 cardiomyocytes	25 µM	24 h co-treatment	Angiotensin II	↓ calmodulin-dependent protein kinase II, Bax, p53, apoptosis	Jin et al. [150]
Gallic acid	Neonatal rat cardiomyocytes	10 µM	24 h angiotensin II followed by unspecified time with gallic acid	Angiotensin II	↓ hypertrophy, MAPK	Yan et al. [151]
Curcumin	Cardiac fibroblasts	5, 10, and 15 µM	1 h pretreatment followed by 12, 24, and 48 h angiotensin II	Angiotensin II	↓ collagen, TGF-β, MMP-2, MMP-9<br>↑ Sirt1	Xiao et al. [188]

Resveratrol	H9c2 cardiomyocytes	20 μM	24 h co-treatment	Hypoxia	↓ apoptosis<br>↑ Sirt1	Chen et al. [184]
Resveratrol	Neonatal rat cardiac myocytes	50 μM	24–48 h co-treatment	Hyperglycemia	↓ O_2^-, Nox subunits, lipid peroxidation<br>↑ cell viability	Guo et al. [94]
Secoisolariciresinol diglucoside	H9c2 cardiomyocytes	500 μM	24 h pretreatment followed by 24 h iron	Iron overload	↓ hypertrophy, H_2O_2, TNF-α, MMP-2 & -9, Bax caspase 3<br>↑ SOD, Bcl-2	Puukila et al. [117]
Resveratrol	H9c2 cardiomyocytes	0, 10, 25, 50 or 75 μM	Resveratrol pretreatment for 24 h followed by doxorubicin for 24 h	Doxorubicin	↓ endoplasmic reticulum stress<br>↑ Sirt1	Lou et al. [95]
Ellagic acid	Rat cardiomyocytes	1–20 μM	18 h co-treatment	Doxorubicin or hypoxia	↓ apoptosis, O_2^-, mPTP, Bnip3<br>↑ mitochondrial membrane potential	Dhingra et al. [139]

↑ denotes an increase, ↓ denotes a decrease. Abbreviations: Akt, protein kinase B; Bax, Bcl-2-associated X protein; Bcl-2, B-cell lymphoma 2; Bnip3, Bcl-2 19-kD interacting protein 3; Ca^{2+}, calcium; Cyt C, cytochrome C; ERK1/2, extracellular signal-regulated kinase; GST1, glutathione S-transferase-1; H_2O_2, hydrogen peroxide; HO-1, heme oxygenase 1; JNK, c-Jun N-terminal kinase; MAPK, mitogen-activated protein kinase; MMP, matrix metalloproteinase; mPTP, mitochondrial permeability transition pore; mTOR, mammalian target of rapamycin, Nox, NADPH-oxidase; NF-κB, nuclear factor kappa-light-chain-enhancer of activated B cells; Nrf2, nuclear factor erythroid 2-related factor 2; NQO1, NADPH quinone dehydrogenase 1; O_2^-, superoxide; PI3K, phosphoinositide 3-kinase; ROS, reactive oxygen species; SERCA2a, sarcoplasmic-endoplasmic reticulum Ca2+ ATPase; Sirt1, sirtuin 1; SOD, superoxide dismutase; TAK1, transforming growth factor-β-activated kinase 1; TGF-β, transforming growth factor-beta; TNF-α, tumor necrosis factor-alpha; ULK1, unc-51-like kinase 1.

Table S2. Effects of Polyphenols in *ex vivo* and *in vivo* preclinical models of HF.

Polyphenol(s)	Subject	Dosage	Duration	Insult	Findings	Author
Anthocyanin rich corn containing: cyanidin-glucoside, cyanidin-malonylglucoside, pelargonidin-malonylglucoside, cyanidin-dimalonylglucoside	Wistar rats	20% of diet	8-week pretreatment	*Ex vivo* ischemia-reperfusion	↓ infarct size<br>↑ antioxidant capacity	Toufektsian et al. [86]
Luteolin	Sprague-Dawley rats	40 μmol/L	30-minute pretreatment	*Ex vivo* ischemia-reperfusion	↓ cardiac function, infarct size	Fang et al. [88]
Vanillic acid	Sprague-Dawley rats	5 and 10 mg/kg	10-day pretreatment	*Ex vivo* ischemia-reperfusion	↓ lipid peroxidation<br>↑ SOD, catalase, GPx, antioxidant capacity	Dianat et al [100]
Cyanidin-3-glucoside, delphinidin-3-glucoside, pelargonidin-3-glucoside	Wistar rats	20 μM	15 min perfusion with anthocyanins prior to ischemia	*Ex vivo* ischemia in isolated heart	↓ caspase-3 activity<br>↑ mitochondrial respiration, cytochrome c reduction	Skemiene et al. [132]
Cyanidin-3-glucoside, delphinidin-3-glucoside, pelargonidin-3-glucoside	Wistar rats	10, 20 or 40 μM	15 min perfusion with anthocyanins prior to ischemia	*Ex vivo* ischemia in isolated heart	↑ mitochondrial respiration, ATP production	Skemiene et al. [133]
Luteolin	Sprague-Dawley rats	200 mg/kg/d	2-week pretreatment	Ischemia-reperfusion	↓ infarct size, Bax, cleaved caspase 3<br>↑ Bcl-2, SERCA2a, Akt	Nai et al. [90]
Daidzein	Sprague–Dawley rats	5 or 10 mg/kg/d	1 h pretreatment	Ischemia-reperfusion	↓ NF-κB, plasma IL-6 and TNF-α, caspase-3 activity<br>↑ cardiac function, catalase	Kim et al. [82]
Genistein	Rabbits	1 mg/kg	5 min pretreatment	Ischemia-reperfusion	↓ infarct size, apoptosis, Bax	Ji et al [83]
(−)-epicatechin	Sprague-Dawley rats	1 mg/kg/d	2- or 10-day pretreatment followed by ischemia-reperfusion with 2 or 21 days before sacrifice, respectively	Ischemia-reperfusion	↓hypertrophy, infarct area, lipid peroxidation, MMP-9 and MMP-2<br>↑Antioxidant capacity	Yamazaki et al. [87]
Chlorogenic acid	Aged SAMP8 mice	10 or 20 mg/kg/d	2-week pretreatment	Ischemia-reperfusion	↓ IL-1β, TNF-α, infarct size, mitochondrial ROS, JNK, SOD2, cytochrome C<br>↑ SOD, GPx, oxygen consumption	Li et al. [134]
Naringenin	Sprague-Dawley rats	50 mg/kg/d	5-day pretreatment	Ischemia-reperfusion	↓ infarct size, apoptosis, cleaved caspase 3, O_2^-, lipid peroxidation, Nox subunit,<br>↑ cardiac function, catalase, SOD2	Yu et al. [78]

Urolithin A	C57BL/6 mice	1 mg/kg	24 h pretreatment	Ischemic reperfusion	↓ apoptosis<br>↑ FS, SOD	Tang et al. [172]
Urolithin B	Sprague-Dawley rats	0.7 mg/kg/d	2-day pretreatment	Ischemia-reperfusion	↓ infarct size, cleaved caspase-3, apoptosis<br>↑ cardiac function, mTOR, ULK1, O_2^-, SOD	Zheng et al. [106]
Secoisolariciresinol diglucoside	Sprague-Dawley rats	20mg/kg/d	8 weeks high-cholesterol diet followed by 2 weeks of secoisolariciresinol diglucoside	Ischemic reperfusion in hypercholesterolemic myocardium	↓ infarct size<br>↑ HO-1, EF, FS	Penumathsa et al. [114]
Apigenin	Wistar rats	10, 20 o 40 mg/kg	24 h pretreatment	Coronary artery ligation	↓ infarct size, MMP-9, TNF-α, IL-1β, IL-6, NF-κB, caspase 3	Du et al. [75]
Hesperetin	C57BL/6 mice	30 mg/kg/d	8-week treatment following surgery	Coronary artery ligation	↓ fibrosis, TNF-α, IL-1β and IL-6, NF-κB<br>↑ EF	Wang et al. [80]
Chlorogenic acid	C57BL/6 mice	30 mg/kg/d	7- or 14-day treatment following surgery	Coronary artery ligation	↓ hypertrophy, infarct size<br>↑ FS	Kanno et al. [109]
Curcumin	C57BL/6J mice	100 mg/kg/d	1-week pretreatment followed by 4 weeks post-surgery	Coronary artery ligation	↓ fibrosis, collagen, TGF-β, infarct size<br>↑ Sirt1	Xiao et al. [188]
Delphinidin	C57BL/6J mice	5 or 15 mg/kg/d	8-week treatment following surgery	Transverse aortic constriction	↓ hypertrophy, fibrosis O_2-, Nox activity<br>↑ EF	Chen et al. [68]
Myricetin	C57BL/6J mice	200 mg/kg/d	6-week treatment following surgery	Transverse aortic constriction	↓ hypertrophy. Fibrosis, MAPK, NF-κB, TAK1<br>↑ EF, FS, Nrf2, HO-1	Liao et al. [74]
Genistein	Kunming mice	10, 50 and 100 mg/kg/d	8-week treatment following surgery	Transverse aortic constriction	↓ hypertrophy, fibrosis<br>↑ EF, FS	Qin et al. [81]
Gallic acid	CD-1 mice	100 mg/kg/day	2-week treatment following HF induction	Transverse aortic constriction	↓ hypertrophy, fibrosis, collagen synthesis<br>↑FS	Jin et al. [101]
Chlorogenic acid	C57BL/6N mice	110 mg/kg/d	4-week treatment following surgery	Transverse aortic constriction	↓ TNF-α<br>↑ EF, FS,	Tian et al. [108]
Gallic acid	C57BL/6 mice	5 or 20 mg/kg/d	8-week treatment following surgery	Transverse aortic constriction	↓hypertrophy, fibrosis, collagen synthesis, MAPK, IL-6, IL-1β, O_2^-, Akt<br>↑FS, EF, ULK1	Yan et al. [151]
Epigallocatechin-3 gallate	Sprague Dawley rats	0.02%, 0.04% and 0.08% in drinking water	3- or 8-week treatment following surgery	Abdominal aortic constriction	↓ hypertrophy, fibrosis<br>↑ cardiac function	Hao et al. [85]

Luteolin	Sprague-Dawley rats	10 µg/kg/d	2-week treatment following surgery	Abdominal aortic constriction	↓ fibrosis, Bax, cleaved caspase 3<br>↑ EF, FS, Bcl-2	Hu et al. [89]
Hesperetin	C57BL/6 mice	30 mg/kg/d	7-week treatment 1 week after surgery	Aortic banding	↓ hypertrophy, JNK, fibrosis, TGF-β1, Nox subunits, apoptosis, Bax, cleaved caspase 3<br>↑ EF, FS, SOD1, SOD2	Deng et al. [76]
Hesperetin	Wistar rats	Not provided	30-day pretreatment followed by 7 days isoproterenol	Isoproterenol	↓ hypertrophy, fibrosis, lipid peroxidation, KEAP1<br>↑ antioxidant capacity, NQO1, HO-1, Nrf2	Velusamy et al. [79]
Ellagic Acid	Wistar Rats	7.5 & 15 mg/kg/d	10-day pretreatment followed by 2 days isoproterenol injections	Isoproterenol	↓ tachycardia, hypertrophy, lipid peroxidation, infarct size	Kannan & Quine [104]
Gallic acid	Wistar and spontaneously hypertensive rats	1% in tap water	4-month co-treatment	Hypertension	↓ blood pressure, hypertrophy, calmodulin-dependent protein kinase II, cleaved caspase-3, Bax, p53	Jin et al. [150]
Resveratrol	double transgenic rats harboring human renin and angiotensinogen genes	800 mg/kg/d	4-week treatment	RAAS-induced cardiac remodeling	↓ hypertrophy<br>↑ survival, Sirt1	Biala et al. [91]
Resveratrol	Sprague-Dawley rats	2.5 mg/kg/d	2 days post-surgery for 26 days or 14 days post-surgery for 14 days	Volume or pressure overload	↑ cardiac function in pressure overload only	Wojciechowski et al [92]
Purple rice extract containing 73% cyanidin-3-glucoside	Wistar rats	250 mg/kg/d	4-week treatment following streptozotocin injection	Streptozotocin	↓ fibrosis, NF-κB, MMP-9, MAPK, TGF-ß	Chen et al. [84]
Urolithin A and B	Wistar rats	2.5 mg/kg/d	3-week treatment following streptozotocin injection.	Streptozotocin	↑ cardiac function, SERCA2a Improved Ca^{2+} flux.	Savi et al. [149]
Anthocyanin rich black rice anthocyanin extract containing ~73% cyanidin-3-glucosude	Wistar rats	250 mg/kg/d	4-week treatment following streptozotocin injection	Streptozotocin	↓apoptosis, cleaved caspase 3<br>↑ Akt, Bcl-xL, Bcl-2, FS	Liu et al. [171]
Secoisolariciresinol diglucoside	Wistar rats	25 mg/kg/d	14-day pretreatment + 21-day treatment with secoisolariciresinol	Monocrotaline-induced right heart dysfunction	↓ hypertrophy, H_2O_2, lipid peroxidation, catalase, SOD, GPx	Puukila et al. [116]

			diglucoside following monocrotaline injection, or 21-day treatment following monocrotaline injection		↑ cardiac function	

↑ denotes an increase, ↓ denotes a decrease. Abbreviations: Akt, protein kinase B; Bax, Bcl-2-associated X protein; Bcl-2, Bcl-xL, B-cell lymphoma 2; B-cell lymphoma-extra-large; Ca^{2+}, calcium; Cyt C, cytochrome C; ERK1/2, extracellular receptor kinase; EF, ejection fraction; FS, fractional shortening; GPx, Glutathione peroxidase; H_2O_2, hydrogen peroxide; HO-1, heme oxygenase 1; IL, interleukin; JNK, c-Jun N-terminal kinase; KEAP1, kelch-like ECH-associated protein 1; MAPK, mitogen-activated protein kinase; MMP, matrix metalloproteinase; mTOR, mammalian target of rapamycin; NF-κB, nuclear factor kappa-light-chain-enhancer of activated B cells; Nox, NADPH-oxidase; Nrf2, nuclear factor erythroid 2-related factor 2; NQO1, NADPH quinone dehydrogenase 1; O_2^-, superoxide; ROS, reactive oxygen species; SERCA2a, sarcoplasmic-endoplasmic reticulum Ca^{2+} ATPase; Sirt1, sirtuin 1; SOD, superoxide dismutase; TAK1, transforming growth factor beta-activated kinase 1; TGF-β, transforming growth factor-beta; TNF-α, tumor necrosis factor-alpha; ULK1, unc-51-like kinase 1.
